# Assessing Caregiver Burden in Kidney Failure: A Systematic Review of Measurement Properties of Instruments

**DOI:** 10.1016/j.xkme.2025.101054

**Published:** 2025-06-24

**Authors:** Ravi Shankar, Eunice Wei Xin Lee, Nan Luo, Priyanka Khatri, Leanne Leong, Amartya Mukhopadhyay, Horng-Ruey Chua, Wei-Zhen Hong

**Affiliations:** 1 Research and Innovation, Medical Affairs, Alexandra Hospital, Singapore; 2 Yong Loo Lin School of Medicine, National University of Singapore, Singapore; 3 Saw Swee Hock School of Public Health, National University of Singapore, Singapore; 4 Fast and Chronic Programmes, Alexandra Hospital, Singapore; 5 Division of Respiratory and Critical Care Medicine, Department of Medicine, National University Hospital, Singapore; 6 Division of Nephrology, Department of Medicine, National University Hospital, Singapore

**Keywords:** Chronic kidney disease, end-stage kidney disease, caregiver burden, psychometrics, patient-reported outcome measures

## Abstract

Caregiver burden in end-stage kidney disease requires assessment using validated instruments. This systematic review evaluated measurement properties of caregiver burden instruments in end-stage kidney disease using Consensus-Based Standards for the Selection of Health Measurement Instruments methodology. We searched 8 databases (PubMed, CINAHL, Embase, Web of Science, MEDLINE, Cochrane Library, Scopus, and PsycINFO) for studies evaluating measurement properties of caregiver burden instruments in caregivers of patients with kidney failure. The Consensus-Based Standards for the Selection of Health Measurement Instruments Risk of Bias checklist assessed methodological quality, and Grading of Recommendations Assessment, Development and Evaluation approach evaluated evidence quality. Twenty-four studies evaluating 21 distinct caregiver burden instruments were included. The Zarit Burden Interview, Caregiver Burden Scale, and Beck Depression Inventory were most frequently evaluated. Only a few instruments had high-quality evidence of sufficient content validity, internal consistency, test-retest reliability, and construct validity. Cross-cultural validity was assessed for only 9 instruments, with most rated as indeterminate owing to methodological limitations. No studies evaluated responsiveness to change over time. Quality of evidence was low to very low for most measurement properties, highlighting significant gaps in validation research. The Zarit Burden Interview, Caregiver Burden Scale, and Beck Depression Inventory show promising validity and reliability but require further testing. Rigorously conducted validation studies, involvement of caregivers of patients with kidney failure in content validation, and cross-cultural adaptations are priorities for optimizing burden assessment in this unique population.

## Introduction

End-stage kidney disease (ESKD) is the final stage of chronic kidney disease characterized by severely diminished kidney function necessitating dialysis or kidney transplantation for survival.^1^ The global prevalence of ESKD continues to increase, driven by population aging and the growing burden of diabetes and hypertension.^2^^,^^3^ Patients with ESKD face a high symptom burden, complex treatment regimens, and impaired quality of life.^4^

The challenges of living with ESKD extend beyond the patient to significantly affect their informal caregivers, often family members, who provide vital unpaid care and support.^5^ Caregivers of patients with ESKD assist with managing the illness, treatment adherence, dietary restrictions, appointments, and medications as well as providing emotional support and performing household tasks the care recipient can no longer do.^6^ The immense physical, emotional, social, and financial toll of providing such care is conceptualized as “caregiver burden.”^7^

Caregivers of patients with ESKD experience high levels of burden as evidenced by impaired mental and physical health, reduced quality of life, loss of social connectedness, and financial strain.^8^ Left unaddressed, caregiver burden can lead to burnout and compromise the sustainability of informal care.^9^ Burden also predicts adverse patient outcomes, including peritonitis, hospitalization, and mortality,^10^ highlighting the negative repercussions of burdened caregivers on ESKD care. Experts increasingly recognize caregiver burden as a priority concern in ESKD.^11^

To effectively identify and address the unique burden experienced by caregivers of patients with kidney failure, reliable and valid instruments are essential. Measurement plays a key role in assessing the prevalence and severity of burden to target supports, evaluating the effectiveness of caregiver interventions, and generating robust evidence to guide policy and practice. The choice of instrument directly affects the quality of burden data and the conclusions drawn from them.^12^ Instruments with poor or unknown measurement properties may fail to detect burden or capture clinically meaningful changes, leading to inappropriate care decisions.

Despite the recognized importance of assessing caregiver burden in ESKD, there is no consensus on the optimal instrument for this population.^13^ A wide range of both generic and ESKD-specific instruments have been used, often without sufficient consideration of their validity, reliability, and appropriateness for the unique ESKD caregiving context.^14^ Systematic reviews have focused on describing available instruments and the prevalence of burden in caregivers of patients with kidney failure^13^ but have not critically evaluated the measurement properties of these instruments against established standards.

The methodological quality of caregiver burden instruments is determined by their measurement properties, the key attributes that reflect the quality, reliability, and validity of an instrument.^15^ These properties are not inherent but must be established in the specific context of use. The Consensus-Based Standards for the Selection of Health Measurement Instruments (COSMIN) initiative has developed widely accepted taxonomy, terminology, and criteria for evaluating measurement properties of health-related patient-reported outcome measures.^15^^,^^16^ Although caregiver burden instruments are not strictly patient-reported outcome measures, the COSMIN framework is still highly relevant as these instruments measure subjective constructs and face similar measurement challenges as patient-reported outcome measures.^17^

A comprehensive and critical appraisal of the measurement properties of caregiver burden instruments used in ESKD is needed to inform optimal instrument selection for research and practice. The objectives of this systematic review were as follows.

1. To identify caregiver burden instruments used in ESKD and their key characteristics.
2. To evaluate and compare the measurement properties of these instruments.
3. To evaluate the methodological quality of the validation studies using the COSMIN Risk of Bias checklist and quality of the evidence using Grading of Recommendations Assessment, Development and Evaluation.
4. To identify gaps in the evidence on measurement properties and provide recommendations for future validation research and instrument selection.

## Methods

The review protocol was registered in the International Prospective Register of Systematic Reviews (registration number: CRD42023433906). The review followed the Preferred Reporting Items for Systematic Reviews and Meta-analyses 2020 reporting guideline.^18^

### Eligibility Criteria

We included studies on informal caregivers of patients with ESKD (stage 5 chronic kidney disease) who were receiving either dialysis or conservative management (without intention for dialysis), in which the studies evaluated and reported at least 1 measurement property of an instrument measuring caregiver burden. Informal caregivers were defined as unpaid family members or friends who provide care to the patient with ESKD. We excluded studies on caregivers of kidney transplant patients, studies using instruments not specific to caregiver burden, and studies published in languages other than English. The exclusion of “other caregiving-related constructs” was deliberate to maintain conceptual focus. These constructs (quality of life, satisfaction, positive aspects of caregiving) measure distinct concepts that, although valuable, would broaden scope beyond caregiver burden’s core definition as a perceived negative impact of caregiving. Similarly, objective measures (eg, hours spent caregiving) were excluded as they do not capture the subjective burden experience central to this review. Table 1 summarizes the inclusion and exclusion criteria.

**Table 1:** Inclusion and Exclusion Criteria

Category	Inclusion Criteria	Exclusion Criteria
Construct of interest	Instruments specifically designed to measure caregiver burden, defined as the perceived negative impact (psychological, social, physical, financial) of providing care	• Instruments primarily measuring other caregiving-related constructs even if subjective (eg, quality of life, satisfaction with caregiving role, positive aspects of caregiving) • Instruments measuring objective burden indicators only (eg, tasks performed, hours of care)
Population of interest	Informal caregivers (family members or friends) providing unpaid care to patients with ESKD (dialysis or conservative management)	Caregivers of transplant patients; formal or paid caregivers; caregivers of patients with earlier stage CKD
Study design	Development or validation study of measurement properties published in English, regardless of the time period or setting	Editorial, letter, commentary, literature review, systematic review, and meta-analysis
Outcomes	Studies evaluating psychometric properties of caregiver burden instruments when used with caregivers of patients with kidney failure, including: - content validity - structural validity - internal consistency - cross-cultural validity/measurement invariance - reliability - measurement error - criterion validity - hypothesis testing for construct validity - responsiveness	• Studies that only use caregiver burden instruments as outcome measures without evaluating their measurement properties - Studies evaluating measurement properties of instruments designed to measure other constructs (eg, quality of life instruments, satisfaction scales) even if used with caregivers of patients with kidney failure

Abbreviations: CKD, chronic kidney disease; ESKD, end-stage kidney disease.

### Search Strategy

A comprehensive search strategy was developed in collaboration with domain experts. We conducted searches across 8 major databases—PubMed, CINAHL, Embase, Web of Science, MEDLINE, Cochrane Library, Scopus, and PsycINFO—from their inception dates up to June 1, 2024. The search used a combination of indexed terms and free-text keywords to encompass the key concepts of “caregiver,” “end-stage kidney disease,” “burden,” “instrument,” and “measurement properties.”

The following search string was used.

([“caregiver” or “caregiving”] and [“burden” or “stress” or “strain” or “distress” or “load” or “fatigue” or “burnout” or “exhaustion” or “overwhelm” or “anxiety” or “depression” or “emotional and strain” or “psychological and distress” or “emotional and burden” or “physical and burden” or “mental and burden” or “hardship” or “challenge” or “difficulty” or “demand” or “responsibility” or “impact” or “pressure” or “stressors”]) and (“kidney and disease” or “advanced and kidney and disease” or “end and stage and kidney and disease” or “haemodialysis” or “dialysis” or “peritoneal and dialysis”).

Reference lists of included studies and relevant reviews were hand searched.

### Study Selection

After retrieving the search results, duplicates were removed, and the remaining articles were independently screened by 2 reviewers using Covidence Systematic Review Software (Veritas Health Innovation).^19^ Screening was conducted in the following 2 stages: first by title/abstract, and then by full text. A set of predefined inclusion criteria, detailed in Item S2, was used to guide selection during the full-text screening stage. These criteria ensured that only studies investigating measurement properties of caregiver burden instruments in ESKD were included. Disagreements between reviewers were resolved through consensus or, when necessary, by consultation with a third reviewer. This rigorous selection process ensured the inclusion of relevant, high-quality studies that met all specified criteria.

### Data Extraction

Two reviewers independently extracted data from included studies using a pilot-tested data extraction form (Item S4). Extracted data included study characteristics (eg, country, design, sample size, and setting), sample characteristics (eg, caregiver and patient demographics, relationships, and dialysis modality), instrument characteristics (eg, name, constructs, item number, response options, scoring, and administration mode), and any information on measurement properties and their evaluation. Results of each measurement property were extracted, including content validity, structural validity, internal consistency, cross-cultural validity/measurement invariance, reliability, measurement errors, criterion validity, hypothesis testing for construct validity, and responsiveness.

### Methodological Quality Assessment

The COSMIN checklist was used to evaluate the methodological quality of studies for each reported measurement property on a 4-point scale (very good, adequate, doubtful, or inadequate). Two reviewers independently rated each study, with disagreements resolved based on consensus.

The COSMIN framework defines the following 3 main domains of measurement properties: reliability, validity, and responsiveness.^15^ Reliability is the degree to which the measurement is free from measurement errors and includes internal consistency, reliability (test-retest, interrater, and intrarater), and measurement errors.^15^ Validity refers to the degree to which an instrument measures the construct(s) it purports to measure and includes content validity (encompassing face validity), construct validity (structural validity, hypothesis testing, and cross-cultural validity), and criterion validity.^15^ Responsiveness is the ability of an instrument to detect change over time in the construct measured.^15^

The COSMIN methodology provides a systematic 4-step procedure for evaluation, which is as follows: (1) identifying which measurement properties were assessed in each study; (2) determining whether item response theory methods were used; (3) evaluating each property according to specific methodological criteria; and (4) assessing the generalizability of findings. This approach enables comprehensive evaluation of each instrument’s measurement properties.^20^

Quality ratings were assigned according to specific criteria. Very good quality indicates all critical methodological requirements were met, with appropriate sample sizes, proper statistical methods, and comprehensive assessment. Adequate quality suggests most critical requirements were met with minor limitations. Doubtful quality indicates important methodological requirements were not met or inadequately described, whereas inadequate quality signifies critical methodological flaws.

An instrument was considered to have good measurement properties if it achieved at least “adequate” ratings on essential properties (content validity, internal consistency, and reliability) with no “inadequate” ratings. The final assessment followed the “worst score counts” principle, in which a study’s lowest score on any relevant criterion determined its overall rating for that property.

### Data Synthesis and Quality Assessment

Two complementary approaches were used to synthesize and evaluate the measurement properties of the included instruments. First, the updated criteria for good measurement properties as defined using COSMIN were applied. Each measurement property for each instrument was rated as sufficient (+), indeterminate (?), or insufficient (-) based on predefined criteria.^16^ This assessment provides a straightforward evaluation of whether each property meets the standards for being considered “good” (Table 2).

**Table 2:** Summary of the Quality Criteria Used to Assess the Measurement Properties of Instruments

Property	Definition	+	?	−
Internal consistency	Degree of correlation between different items on the same scale	Cronbach alpha(s) ≥ 0.70	Dimensionality not known OR Cronbach alpha not determined	Cronbach alpha(s) < 0.70
Reliability	Consistency and stability of measurements over time and across different raters	ICC/weighted kappa ≥ 0.70, or Pearson r ≥ 0.80	Neither ICC/weighted kappa nor Pearson r determined	ICC/weighted kappa < 0.70 or Pearson r < 0.80
Measurement error	Degree to which observed scores deviate from true scores	MIC > SDC or MIC outside LOA	MIC not defined	MIC ≤ SDC or MIC equals or inside LOA
Content validity	Extent to which a measure captures all relevant aspects of the construct being measured	All items relevant and comprehensive	Not enough information available	Not all items relevant or comprehensive
Structural validity	Extent to which a measure captures all relevant aspects of the construct being measured	Factors explain ≥50% of variance	Explained variance not mentioned	Factors explain <50% of variance
Structural validity (IRT methods)	Extent to which a measure reflects the underlying structure of the construct being measured	Good IRT model fit and no DIF	Important characteristics not reported	Poor IRT model fit or important DIF
Hypothesis testing	Ability of a measure to discriminate between groups that are expected to differ on the construct being measured	Correlation with same construct ≥ 0.50 or ≥75% hypotheses confirmed and correlation with related constructs higher than unrelated constructs	Solely correlations with unrelated constructs	Correlation with same construct < 0.50 or < 75% hypotheses confirmed or correlation with related constructs lower than unrelated constructs
Cross-cultural validity	Extent to which a measure can be used in different cultures and languages	No differences in factor structure or no important DIF between language versions	Multiple group factor analysis not applied and DIF not assessed	Differences in factor structure or important DIF between language versions
Responsiveness	Ability of a measure to detect change over time	Correlation with changes ≥ 0.50 or ≥75% hypotheses confirmed or AUC ≥ 0.70 and correlations with related changes higher than unrelated	Solely correlations with unrelated constructs	Correlations with changes < 0.50 or <75% hypotheses confirmed or AUC < 0.70 or correlations with related changes lower than unrelated
Interpretability	Ease of understanding and communicating the meaning of a measure	MIC calculated, anchor questions described	MIC calculated, anchor questions unclear	MIC not reported
Reproducibility	Degree of agreement between repeated measurements	ICC ≥ 0.70 and SDC or LOA < MIC	ICC or SDC/LOA not reported	ICC < 0.70 or SDC or LOA > MIC
Floor and ceiling effects	Extent to which a measure captures lowest and highest possible scores	≤15% of respondents achieved the lowest or highest possible scores	Percentage of respondents achieving lowest/highest scores not reported	>15% of respondents achieved the lowest or highest possible scores

*Note*: “+” indicates sufficient; “?” indicates indeterminate; and “-” indicates insufficient.

Abbreviations: DIF, differential item functioning; ICC, intraclass correlation coefficient; IRT, item response theory; LOA, limits of agreement; MIC, minimal important change; SDC, smallest detectable change.

Second, the overall quality of evidence for each measurement property of each instrument was graded using a modified Grading of Recommendations Assessment, Development and Evaluation approach. This approach considers factors such as study design, risk of bias, consistency of results, and directness of evidence. The quality of evidence was categorized as high, moderate, low, or very low, reflecting the level of confidence in the assessment of each measurement property.^21^

High quality indicates there is high confidence that the true measurement property lies close to the estimate, with negligible risk of bias in the body of evidence. Moderate quality suggests moderate confidence in the measurement property estimate, in which the true property is likely close to the estimate but with possibility of substantial difference existing, and the evidence has minor concerns regarding risk of bias or inconsistency. Low quality indicates limited confidence in the measurement property estimate, in which the true property may be substantially different from the estimate owing to major concerns regarding risk of bias, inconsistency, or imprecision in the body of evidence. Very low quality reflects very little confidence in the measurement property estimate, with the true property likely substantially different from the estimate owing to very serious concerns regarding risk of bias, inconsistency, imprecision, or indirectness. These quality levels assess the methodological quality and risk of bias in studies evaluating measurement properties, with the assigned level determining the strength of evidence provided by that study and subsequently influencing the overall assessment of the measurement properties.

## Results

### Search Results

**Figure 1:**
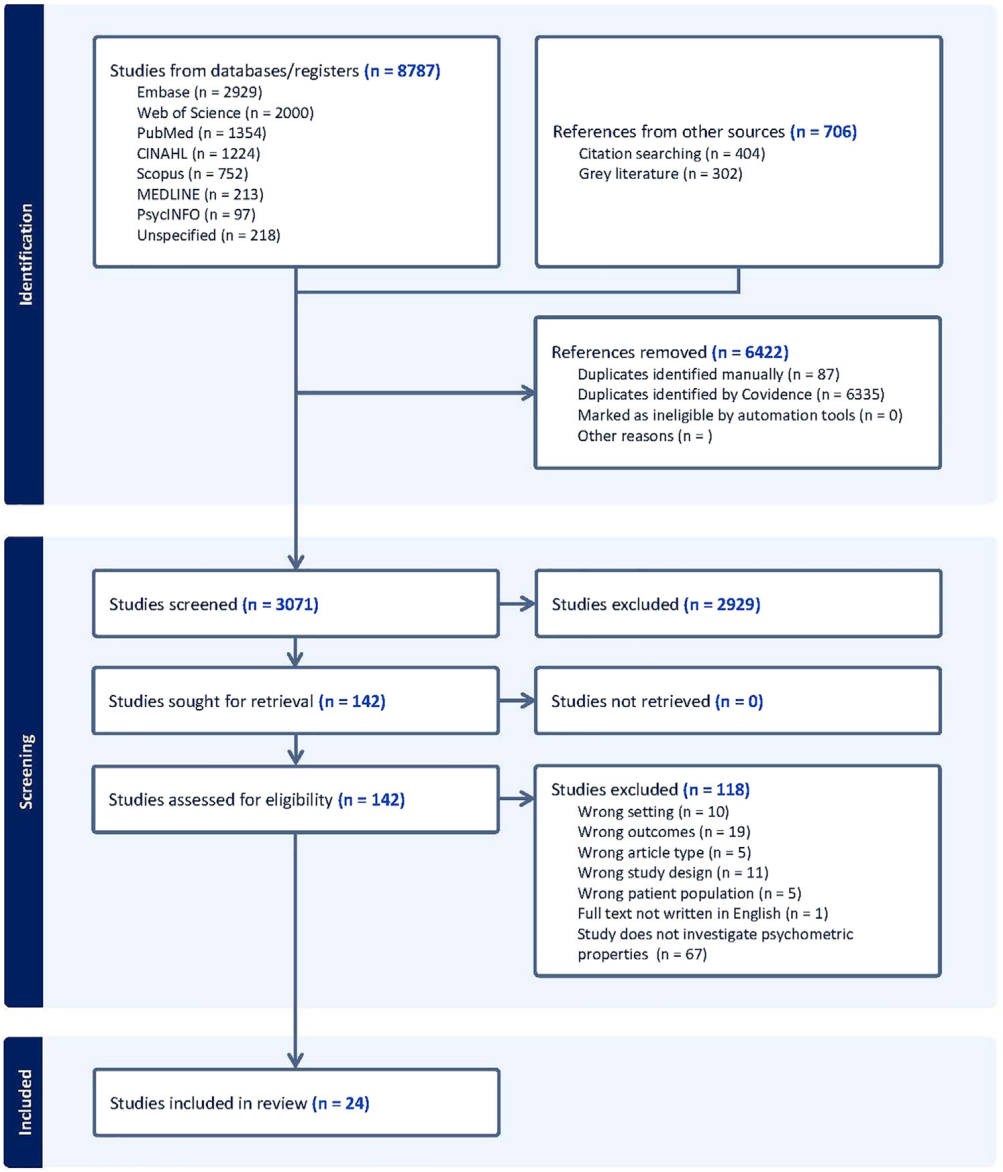
Preferred Reporting Items for Systematic Reviews and Meta-analyses diagram.

The systematic search across 8 databases yielded a total of 8,787 records. From citation searching and gray literature, there were 706 additional records. After removing 6,422 duplicates, 3,071 unique records remained for screening. The title and abstract screening process eliminated 2,929 records that did not meet the inclusion criteria, leaving 142 articles for full-text review. During the full-text screening, 118 articles were excluded for the following reasons: (1) wrong setting (n = 10); (2) wrong outcome (n = 19); (3) wrong article type (n = 5); (4) wrong study design (n = 11); (5) wrong patient population (n = 5); (6) full text not written in English (n = 1); and not investigating the psychometric properties of a caregiver burden instrument (n = 67). After this rigorous selection process, 24 studies met all inclusion criteria and were included in the final review. These studies collectively evaluated 21 distinct caregiver burden instruments used in the context of ESKD. The study selection process is visually represented in the Preferred Reporting Items for Systematic Reviews and Meta-analyses flow diagram (Fig 1).

### Study Characteristics

Table 3 summarizes the key characteristics of included studies. The 24 studies were from 12 countries spanning North America, South America, Europe, the Middle East, and Asia. Most studies used cross-sectional designs, with only 2 longitudinal studies. The total sample size across all studies was 3,872 caregivers, with individual study samples ranging from 50-620 participants (median: 124). Sample size distribution showed 29.2% of studies with fewer than 100 participants, 33.3% with 100-200 participants, and 37.5% with more than 200 participants. Notable larger studies included those conducted by Hejazi et al^22^ (n = 620), Horsburgh et al^23^ (n = 447), Kian et al^24^ (n = 400), and Kosar Sahin et al^25^ (n = 404) (Table 4).^25^, ^26^, ^27^, ^28^, ^29^, ^30^, ^31^, ^32^, ^33^, ^34^, ^35^, ^36^, ^37^, ^38^, ^39^, ^40^, ^41^, ^42^, ^43^, ^44^, ^45^ Demographically, caregivers were predominantly women across all studies (54%-100%), with mean ages ranging from 32-70 years. Relationship patterns showed that caregivers were most commonly spouses (26%-80%) or adult children (6%-56%), with some studies focusing on parents (6%-56% in pediatric-focused research) and siblings (5.6%-45.3%). The average caregiving duration, when reported, ranged from 3.02-6.16 years. Regarding treatment modalities of care recipients, studies focused on caregivers of patients receiving hemodialysis only (45.8%), peritoneal dialysis only (16.7%), or a combination of both modalities (37.5%). No studies specifically addressed caregivers of patients receiving conservative management without dialysis. This substantial and diverse caregiver sample enhances the generalizability of findings, although the considerable variation in sample characteristics across studies should be carefully considered when interpreting results for specific caregiver populations.

**Table 3:** Characteristics of Included Studies

Characteristic	No. of Studies	Percentage
Total studies	24	100
Study design		
Cross-sectional	12	50
Methodological/scale development	6	25
Mixed methods	3	12.5
Other	3	12.5
Country		
Turkey	4	16.7
United States	3	12.5
Iran	3	12.5
Egypt	2	8.3
Spain	2	8.3
Other (single studies)	10	41.7
Sample size		
<100	7	29.2
100-200	8	33.3
201-300	3	12.5
>300	6	25
Types of dialysis		
Hemodialysis only	11	45.8
Peritoneal dialysis only	4	16.7
Both HD and PD	9	37.5

Abbreviations: HD, hemodialysis; PD, peritoneal dialysis.

**Table 4:** Key Study and Sample Characteristics

Author (Year)	Country	Setting	Study Design	Sample Size	Caregiver Age (y) Mean (SD), Range	% Female	Relationship With Patient	Patient Age (y) Mean (SD), Range	Patient Treatment
Alaryni et al (2024)^26^	Saudi Arabia	Not specified	Cross-sectional	50	Majority 31-50, NR	74	Not reported	NR	HD and PD
Albayrak et al (2024)^27^	Turkey	4 Hospitals	Methodological	215	38.1% aged 41-50, NR	64.2	62.8% Mother, 33% father	NR, 5-18	38.6% PD, 33% HD, 1.9% home HD
Alnazly et al (2016)^28^	Jordan	3 Dialysis units	Cross-sectional	139	32.23 (11.78), 21-65	53	26% Spouse, 41% child, 25% other	54.87 (14.01), 18.0-80.0	HD
Arechabala et al (2012)^29^	Chile	5 Dialysis centers	Validation study	161	56 (14.9), NR	40	All blood relation, majority spouses	NR	HD
Ashktorab et al (2017)^30^	Iran	Not specified	Cross-sectional	212	54.2 (13.7), 31-65	100	Wives of patients	NR	HD
Cantekin et al (2016)^31^	Turkey	Not specified	Descriptive	114	HD: 38.24 (12.3); PD: 36.64 (15.08), NR	HD: 31; PD: 34	Not specified	NR	HD and PD
Cil Akinci et al (2014)^32^	Turkey	Not specified	Methodological	161	45.4 (15.3), NR	65.2	50.3% Spouse, 15.5% mother	NR	HD
Cousineau et al (2003)^33^	Canada	Not specified	Cross-sectional	100	64.7 (NR), NR	45	61% Spouse, 16% daughter, 6% son	NR	HD
Darwish et al (2020)^34^	Egypt	Hospital/clinics	Cross-sectional	250	NR	NR	Mothers were main caregivers	11.9 (3.1), NR	85.6% Conservative, 14.4% HD
Feeg et al (2018)^35^	United States	Not specified	Mixed methods	Stage 1: 104; Stage 2: 140; Stage 3: 15	18 to 48+, NR	Stage 1: 71	Parents	NR	Not specified (ESRD included)
Hejazi et al (2022)^22^	Iran	Not specified	Exploratory sequential mixed	620	37.95 (NR), NR	63.5	56.1% Child, 18.9% spouse	NR	HD
Horsburgh et al (2008)^23^	Canada	Not specified	Cross-sectional	447	58.8 (14.3), 18-92	73	Not specified	NR	65% HD, 24% PD, 2% self-care HD
Kian et al (2024)^24^	Iran	Not specified	Methodological	400	40.93 (12), NR	50.5	52% Child, 18.8% spouse	69.22 (6.98), >60	HD
Mahmoud et al (2021)^36^	Egypt	Not specified	Cross-sectional	60	Cases: 38.50 (NR), 20-50 Controls: 40.73 (NR), 23-55	Cases: 90 Controls: 73.3	Mostly parents and family members	NR	Dialysis
Nasim et al (2023)^37^	Pakistan	Hospital	Cross-sectional	124	40.18 (12.26), NR	100	44.64% Spouses, 23.38% parents, 18.54% in laws	NR	HD
Parham et al (2016)^38^	United Kingdom	Not specified	Mixed methods	16 CG, 10 HCP	20-59, NR	87.5	Not specified	NR	Predialysis, PD, HD
Rabiei et al (2021)^39^	Iran	Not specified	Mixed methods	300	42.13 (NR), NR	41	Not specified	NR	HD
Kosar Sahin et al (2024)^25^	Turkey	Not specified	Methodological	404	42.86 (8.12), NR	54.2	45.3% Siblings, 29.0% spouses	NR	HD
Schneider (2003)^40^	United States	Not specified	Cross-sectional	99	60.45 (16), 23-88	82.8	Not specified	62.60 (21.74), 0.80-92.00	HD
Schneider (2004)^41^	United States	Not specified	Cross-sectional	80	62.6 (15.3), NR	81.3	80% Spouse	69.3 (12.5), NR	HD
Sousa et al (2024)^42^	Portugal	Not specified	Cross-sectional	106	52 (16.1), 19-85	80.2	43.4% Spouse, 56.6% other	NR	HD
Tao et al (2020)^43^	China	Not specified	Cross-sectional	60 dyads	59.7 (NR), NR	66.7	60% Spouse, 36.7% adult child	70.7 (NR), >60	PD
Teixidó et al (2006)^44^	Spain	Not specified	Questionnaire development	63	53.43 (12.3), NR	86.4	79.4% Spouse, 11.1% parent	NR	PD
Teixidó-Planas et al (2018)^45^	Spain	Multicenter	Observational	107 dyads	57.50 (14.69), NR	83.2	77.6% Spouses, 15.9% children	63.49 (13.29), NR	PD

*Note:* The table provides an overview of the research landscape, detailing the geographic distribution, study designs, sample sizes, and demographics of both caregivers and patients.

Abbreviations: CG, caregivers; ESKD, end-stage kidney disease; ESRD, end-stage renal disease; HCP, health care professionals; HD, hemodialysis; NR, not reported; PD, peritoneal dialysis.

### Characteristics of Instruments

This systematic review identified 21 distinct instruments used in the context of assessing caregiver burden in caregivers of patients with ESKD. Table 5 presents detailed characteristics of these instruments. The most frequently applied instruments were the Zarit Burden Interview (ZBI) and its short form (ZBI-12) (4 studies), Caregiver Burden Scale (CBS) (4 studies), and Beck Depression Inventory (BDI) (2 studies).

**Table 5:** Characteristics and Psychometric Properties of Caregiver Burden Instruments

Instrument Name	No. of Items	Response Format	Administration Method	Internal Consistency	Test-Retest Reliability	Content Validity	Construct Validity	Structural Validity	Hypothesis Testing	Cross-cultural Validity	Interpretability
ZBI	22	5-Point Likert scale (0-4)	Self-reported	Apha = 0.90-0.93	ICC = 0.89	NR	Supported	Supported by CFA	Supported	Indeterminate	Higher scores indicate higher burden
CBS	22	1 (not at all) to 4 (frequently)	Self-reported	Apha = 0.91 (overall), 0.61-0.83 (subdimensions)	ICC = 0.92	Sufficient	Supported	5-Factor structure confirmed by CFA	Supported	Sufficient for Arabic version	Higher scores indicate higher caregiver burden
BDI	21	Likert scale	Self-reported questionnaires	Alpha = 0.91	r = 0.93	NR	Supported	NR	Supported	Indeterminate	0-13 Minimal, 14-19 mild, 20-28 moderate, 29-63 severe
HD-DT-C	Single-item barometer + 30-item checklist	Barometer: 0-10 scale Checklist: categorical	Self-reported	NR	ICC = 0.991 for barometer, kappa ≥ 0.80 in 77% of checklist items	Sufficient	Supported	NR	Supported	Indeterminate	≥6 fFor research, ≥5 for clinical practice
COPE Index	15	4-Point scale	Interviewer administered	Alpha = 0.714 (entire scale), 0.655-0.864 (subscales)	NR	Sufficient	NR	3 Components extracted by PCA	NR	Indeterminate	Higher scores indicate higher level of perceived positive and negative experience and support quality
PR-CBS	52	5-Point scale	NR	Alpha = 0.911	0.832 (ICC)	Sufficient	NR	Supported by factor analysis	NR	NR	Higher scores indicate higher perceived care tension
PCTQHFC	NR	NR	Self-reported questionnaires	NR	NR	Sufficient (CVR = 0.92)	NR	Supported by CFA	NR	NR	NR
HD-PCBS	34	4-Point Likert scale	Self-reported	Alpha = 0.808-0.901	NR	Sufficient	NR	Unidimensionality confirmed	NR	NR	Higher scores indicate greater burden
LC-GAD	41 (27 on Think subscale, 14 on Task subscale)	5-Point Likert scale	NR	Think LC-GAD: 0.95 Task LC-GAD: 0.83	Think LC-GAD: 0.80 Task LC-GAD: 0.76	NR	Supported	Two-factor structure	Supported	NR	Higher scores indicate higher frequencies of caregiving activities
OQCPPD	NR	NR	NR	Apha = 0.886-0.894	r = 0.512 for overload scale	NR	NR	Unidimensionality confirmed	NR	NR	Higher scores indicate greater overload/impact
SPBS	25	5-Point Likert scale	Interview-administered	Apha = 0.91	NR	Sufficient	NR	Unidimensional scale structure	NR	Indeterminate	Higher scores indicate higher perceived caregiver burden
ZCBS	22	5-Item Likert scale from “never” to “nearly always”	Self-reported	Alpha = 0.92	0.9	NR	NR	Supported by 1 dominant factor	NR	Indeterminate	0-20: little/no burden; 21-40: mild/moderate; 41-60: moderate/severe; 61-88: severe burden
PR-CBS (short version)	20	NR	NR	Alpha = 0.94 (overall scale); 0.86-0.93 (subscales)	ICC = 0.92 (overall scale)	Sufficient	NR	5 Factors explaining 81.73% variance, CFA confirmed 5-factor structure	NR	NR	NR
ZBI-A	12	0-4 Point scale for each item	NR	Alpha = 0.664	NR	NR	NR	NR	NR	Indeterminate	No to mild burden: 0-10; mild to moderate burden: 10-20; high burden: >20
Brief COPE	28	4-Point Likert scale	Self-reported	Alpha = 0.77 for total scale; 0.7-0.91 for subscales	NR	NR	NR	NR	NR	Indeterminate	NR
OCBS	15	5-Point Likert	Self-reported	Alpha = 0.9	NR	NR	NR	Two-factor structure identified and confirmed	Correlations between burden and coping scales assessed	NR	NR
CBQ	21	5-Point Likert scale	Personal interviews	NR	NR	NR	NR	4 factors confirmed in CFA	NR	NR	NR
PNS	36 items	5-Point Likert scale from 0 (not at all) to 4 (extremely)	Self-reported	NR	NR	Sufficient	NR	Two-factor structure identified	NR	NR	Higher scores indicate greater parental needs in that area
PedsQL FIM-A	NR	NR	NR	Alpha > 0.8 for all summary and scale scores	NR	NR	NR	NR	Significant differences found between expected subgroups	Indeterminate	Higher scores indicate better functioning/less negative impact
CPS	8 items originally, 7 items in final Farsi version	5-Point scale from 0 (not at all prepared) to 4 (very well prepared)	Self-reported	Alpha = 0.956; McDonald omega = 0.956	ICC = 0.76	Sufficient	NR	1 factor explaining 75.7% variance, CFA showed acceptable fit	NR	Sufficient	Higher scores indicate higher caregiver preparedness
FSS	9	7-Point Likert scale	Self-reported	Alpha = 0.94	NR	NR	Supported	NR	Supported	Indeterminate	Higher scores indicate greater fatigue severity

Abbreviations: Alpha, Cronbach alpha; CFA, confirmatory factor analysis; CVI, Content Validity Index; EFA, exploratory factor analysis; ICC, intraclass correlation coefficient; NA, not applicable; NR, not reported; PCA, principal component analysis.

Twelve instruments were originally developed for caregivers of non-ESKD populations including ZBI, CBS, BDI, Carers of Older People in Europe (COPE) Index, Self-Perceived Burden Scale (SPBS), Zarit Caregiver Burden Scale (ZCBS), Arabic Abridged version of Zarit Burden Interview (ZBI-A), Brief COPE (Persian translation), Oberst Caregiving Burden Scale (OCBS), Parent Needs Scale (PNS), Caregiver Preparedness Scale (CPS), and Fatigue Severity Scale.

Nine instruments were developed for caregivers of patients with ESKD and include the following: Hemodialysis Distress Thermometer for Caregivers (HD-DT-C), Paediatric Renal Caregiver Burden Scale (PR-CBS), Perceived Care Tension Questionnaire for Caregivers of Hemodialysis Patients (PCTQHFC), Primary Caregiver Burden Scale Individuals Receiving Hemodialysis Treatment (HD-PCBS), Lay Care-Giving for Adults Receiving Dialysis (LC-GAD), Overload Questionnaire for Caregivers of Patients on Peritoneal Dialysis (OQCPPD), PR-CBS short version, Caregiver Burden Questionnaire for Family Caregivers of Hemodialysis Patients (CBQ), and PedsQL Family Impact Module Arabic version (PedsQL FIM-A).

The number of items in these instruments varied widely, ranging from 8 items in the shortest (CPS) to 52 items in the longest (PCTQHFC). Most instruments used Likert-type scales for response options, with the number of response levels ranging from 3-7. Some instruments, such as HD-DT-C, used a combination of scales, featuring a single-item barometer (0-10 scale) and a 30-item categorical checklist.

Recall periods, in which specified, ranged from the present moment to the past month. Scoring approaches varied across instruments, including summing item responses, averaging item responses, or transforming scores to a 0-100 scale. Score interpretation also differed, with only some instruments having established cutoff scores. For example, ZBI uses cutoffs to categorize burden levels (0-20: little/no burden; 21-40: mild/moderate; 41-60: moderate/severe; and 61-88: severe burden).

Administration methods included self-report, interview-administered, and mixed methods. Completion time, in which reported, ranged from 5-30 minutes. For instance, LC-GAD can be completed in 15 minutes with a grade 5 reading level, whereas CBS takes an average of 30 minutes to complete.

Some instruments, such as PR-CBS and HD-PCBS, were developed with extensive input from caregivers and health care professionals, enhancing their content validity for the ESKD population. Cross-cultural adaptations were reported for several instruments, including Arabic versions of ZBI, CBS, and OCBS, and a Turkish version of PR-CBS.

### Results on Measurement Properties

Table 6 provides the evidence synthesis on measurement properties per instrument.

**Table 6:** Quality Assessment of Caregiver Burden Instruments in ESKD: Measurement Properties and Evidence Strength

Instrument	Internal Consistency	Reliability	Content Validity	Structural Validity	Hypothesis Testing	Cross-cultural Validity
ZBI	+/Moderate	+/Moderate	NR	+/Low	+/Low	?/Very low
CBS	+/Moderate	+/Moderate	+/Moderate	+/Moderate	+/Low	+/Moderate (Arabic)
BDI	+/Moderate	+/Moderate	NR	NR	+/Low	?/Very low
HD-DT-C	NR	+/Moderate	+/Moderate	NR	+/Low	?/Very low
COPE Index	?/Low	NR	+/Moderate	+/Low	NR	?/Very low
PR-CBS	+/Moderate	+/Moderate	+/Moderate	+/Moderate	NR	NR
PCTQHFC	NR	NR	+/Moderate	+/Low	NR	NR
HD-PCBS	+/Moderate	NR	+/Low	+/Low	NR	NR
LC-GAD	+/Moderate	+/Low	NR	+/Low	+/Low	NR
OQCPPD	+/Low	?/Low	NR	+/Low	NR	NR
SPBS	+/Moderate	NR	+/Low	+/Low	NR	?/Very low
ZCBS	+/Moderate	+/Moderate	NR	+/Low	NR	?/Very low
PR-CBS (short version)	+/Moderate	+/Moderate	+/Moderate	+/Moderate	NR	NR
ZBI-A	?/Low	NR	NR	NR	NR	?/Very low
Brief COPE	+/Low	NR	NR	NR	NR	?/Very low
OCBS	+/Moderate	NR	NR	+/Low	?/Low	NR
CBQ	NR	NR	NR	+/Low	NR	NR
PNS	NR	NR	+/Moderate	+/Low	NR	NR
PedsQL FIM-A	+/Moderate	NR	NR	NR	+/Low	?/Very low
CPS	+/High	+/Moderate	+/High	+/Moderate	NR	/Moderate
FSS	+/Moderate	NR	NR	NR	+/Low	?/Very low

*Notes:* Measurement properties of instrument: ”+” indicates sufficient; “?” indicates indeterminate; “-” indicates insufficient. Quality of evidence: high, moderate, low, and very low.

Abbreviations: ESKD, end-stage kidney disease; NR, not reported.

#### Content Validity

Content validity, considered the most important property according to COSMIN, was rated as sufficient (+) for the following 6 instruments: HD-DT-C, PR-CBS, PCTQHFC, CBS, PNS, and CPS.^21^ These assessments were based on qualitative research with the target population (such as cognitive interviewing and focus group interviews) and quantitative methods (eg, content validity indexing).

For instance, the content validity for HD-DT-C was assessed using the Item-Content Validity Index and focus group interviews. PR-CBS underwent extensive caregiver and professional input, as well as qualitative assessment by experts and quantitative evaluation using the content validity ratio. PCTQHFC demonstrated high content validity with a scale-level content validity ratio of 0.92. CBS showed strong content validity with the Item-Content Validity Index ranging from 0.89 to 1.00 and a Scale-Content Validity Index of 0.89. PNS was reviewed by expert panels in pediatric nephrology. CPS showed robust content validity with a content validity ratio and kappa coefficient of >0.75 for all items, and a Scale-level Content Validity Index of 1.

However, the studies had doubtful or inadequate methodological quality. Other instruments such as the COPE Index, OQCPPD, HD-PCBS, and SPBS reported content validity assessment through expert reviews or previous studies but provided less-detailed information. ZCBS and PR-CBS reported content validity but did not provide specific indices. Overall, although most instruments demonstrated efforts to establish content validity, the quality and extent of evidence varied across the tools. The remaining instruments either had indeterminate (?) or no evidence of content validity in caregivers of patients with kidney failure.

#### Structural *Validity*

Structural validity was evaluated for 13 instruments (COPE Index, PR-CBS, PCTQHFC, OQCPPD, HD-PCBS, LC-GAD, CBS, SPBS, ZCBS, PR-CBS, OCBS, PNS, and CPS). It was rated as sufficient (+) for 11 instruments. Six instruments (PR-CBS, PCTQHFC, CBS, PR-CBS, PNS, and CPS) demonstrated sufficient structural validity based on confirmatory factor analysis (CFA), whereas 5 (COPE Index, LC-GAD, SPBS, ZCBS, and OCBS) were supported by exploratory factor analysis.

For example, CBS showed a 5-factor structure confirmed using CFA. PR-CBS demonstrated a 5-factor structure explaining 81.73% of the variance in exploratory factor analysis, which was then confirmed using CFA. The COPE Index identified 3 factors through exploratory factor analysis.

However, most studies had doubtful methodological quality owing to small sample sizes or unclear reporting. OQCPPD and HD-PCBS reported unidimensionality but provided limited details on their analyses. CPS stood out with a more comprehensive analysis, showing a one-factor structure explaining 75.7% of variance in exploratory factor analysis and providing detailed CFA fit indices (comparative fit index = 0.991, goodness-of-fit index = 0.969, Tucker–Lewis index = 0.982, root mean square error of approximation = 0.077).

#### Internal Consistency

Internal consistency was evaluated and deemed sufficient (+) for majority of the instruments, as indicated by a Cronbach alpha coefficient of >0.70. Fifteen instruments demonstrated good to excellent internal consistency with a Cronbach alpha coefficient of ≥0.80, whereas 3 (COPE Index, OCBS, and ZBI-A) showed acceptable internal consistency with a Cronbach alpha coefficient between 0.70 and 0.79.

For example, the Fatigue Severity Scale showed excellent internal consistency with a Cronbach alpha coefficient of 0.94. PR-CBS demonstrated high internal consistency with a Cronbach alpha coefficient ranging from 0.911-0.93 for its subscales. CBS reported a Cronbach alpha coefficient of 0.91 for the overall scale and 0.61-0.83 for its subdimensions.

However, some studies had doubtful methodological quality owing to small sample sizes or unclear reporting of methods. The COPE Index reported a lower Cronbach alpha coefficient of 0.714 for the entire scale, whereas its subscales ranged from 0.655-0.864. CPS provided the most comprehensive analysis, reporting not only Cronbach alpha (0.956) but also McDonald omega (0.956) and average interitem correlation (0.756).

#### Cross-cultural Validity

Cross-cultural validity was evaluated for only 9 instruments (HD-DT-C, BDI, ZBI, SPBS, CBS, ZCBS, ZBI-A, Brief COPE, and PedsQL FIM-A), with most rated as indeterminate (?) owing to methodology limitations. Only CBS and CPS had high-quality evidence of sufficient cross-cultural validity. CBS underwent rigorous forward-backward translation from English to Arabic and was validated in this population. CPS was translated and culturally adapted to Farsi using guidelines by Beaton et al,^46^ which are considered robust for cross-cultural adaptation. The remaining instruments either lacked detailed information on their cross-cultural validation process or had limited assessment of their measurement properties in the adapted versions, highlighting a significant gap in the cross-cultural evaluation of caregiver burden instruments in ESKD research. Several instruments underwent translation and cultural adaptation processes including PR-CBS, HD-DT-C, CBS, SPBS, ZCBS, Brief COPE, ZBI-A, PedsQL FIM-A, CPS, and the COPE Index. PR-CBS was validated in a Turkish version. HD-DT-C was translated to American English. CBS underwent forward-backward translation from English to Turkish. SPBS was validated in a Spanish translation. ZCBS was translated and culturally adapted for use in Turkey. Brief COPE underwent translation and cultural adaptation for use in Iran. ZBI-A and PedsQL FIM-A were validated for use in Arabic-speaking populations. CPS was translated and culturally adapted to Farsi. The COPE Index was adapted for use in Pakistan. These adaptations aim to ensure that these instruments remain valid and reliable across different linguistic and cultural contexts, enhancing their use in global health-related quality of life research for caregivers of chronic kidney disease patients. PNS was originally developed in Hong Kong and then modified for use in the United States.

#### Test-Retest Reliability

Test-retest reliability was evaluated for 11 instruments (HD-DT-C, COPE Index, PR-CBS, PCTQHFC, BDI, ZBI, OQCPPD, LC-GAD, CBS, ZCBS, and CPS). It was rated as sufficient (+) for 8 instruments. Seven instruments (HD-DT-C, PR-CBS, BDI, ZBI, OQCPPD, CBS, and ZCBS) demonstrated good to excellent test-retest reliability with intraclass correlation coefficients (ICC) or Pearson r ≥ 0.80, whereas 1 (CPS) showed acceptable test-retest reliability with ICC = 0.76.

For example, HD-DT-C showed excellent test-retest reliability with ICC = 0.991 for the barometer and kappa ≥ 0.80 in 77% of checklist items. ZBI demonstrated high reliability with ICC = 0.89. CBS reported strong test-retest reliability with ICC = 0.92 for the overall scale.

However, some studies had doubtful methodological quality owing to small sample sizes or unclear reporting of time intervals between tests. LC-GAD reported lower test-retest reliability coefficients (r = 0.80 for Think LC-GAD and r = 0.76 for Task LC-GAD). OQCPPD showed moderate test-retest reliability (r = 0.512) for its overload scale. The COPE Index and PCTQHFC did not report specific test-retest reliability coefficients. This highlights the need for more comprehensive and standardized reporting of test-retest reliability across caregiver burden instruments in ESKD research.

#### Measurement Error

Measurement error was evaluated for only one instrument, CBQ, which reported a standard error of measurement of 1.39; no further context or interpretation was provided for this value. The study had doubtful methodological quality owing to unclear reporting and lack of comparison with a minimally important change value.

#### Construct Validity (Hypothesis Testing)

Construct validity (hypothesis testing) was evaluated for 13 instruments (Fatigue Severity Scale, HD-DT-C, COPE Index, ZBI, PR-CBS, BDI, OQCPPD, HD-PCBS, LC-GAD, CBS, ZCBS, OCBS, and PedsQL FIM-A). It was rated as sufficient (+) for 11 instruments. Nine instruments demonstrated expected correlations with related constructs, whereas 2 (ZBI and SRRS) showed significant associations with depression and burden in caregivers through logistic regression.

For example, HD-DT-C showed strong correlations (r ≥ 0.50) in expected directions with Hospital Anxiety and Depression Scale, World Health Organization Quality of Life–Brief Version, and ZBI. CBS demonstrated negative correlations with SF-36 quality of life scores and positive correlations with the Maslach Burnout Scale. PedsQL FIM-A showed significant differences between expected subgroups, supporting its construct validity.

However, most hypothesis-testing studies had doubtful or inadequate quality ratings owing to lack of a priori hypotheses. The COPE Index and PR-CBS reported examining correlations with related measures but did not provide specific correlation coefficients. OCBS assessed correlations between burden and coping scales but did not report detailed results.

#### Criterion Validity

No studies evaluated criterion validity against a “gold standard,” because no gold standard exists for caregiver burden in ESKD.

#### Responsiveness

No studies evaluated responsiveness to change over time for any of the instruments, highlighting a significant gap in the current evidence base.

### Overall Quality of Evidence

This systematic review of caregiver burden instruments in ESKD shows a complex landscape of evidence quality across various measurement properties as shown in Table 6. Notably, high-quality evidence was not found for any instrument. This underscores a significant gap in the current research landscape and highlights the pressing need for more rigorous studies in this field.

Moderate-quality evidence was more prevalent, particularly for internal consistency, reliability, and structural validity across several instruments. ZBI, CBS, BDI, and PR-CBS demonstrated moderate-quality evidence for internal consistency and reliability. CBS and PR-CBS also showed moderate-quality evidence for content validity and structural validity. CPS exhibited moderate-quality evidence across multiple properties, including reliability, structural validity, and cross-cultural validity. This suggests that these instruments have a stronger evidence base compared with others, particularly for these specific properties.

Low-quality evidence was common across many instruments and properties. Structural validity often had low-quality evidence, as seen in the COPE Index, PCTQHFC, HD-PCBS, LC-GAD, OQCPPD, SPBS, ZCBS, OCBS, CBQ, and PNS. Hypothesis testing also frequently showed low-quality evidence, as observed in ZBI, CBS, BDI, HD-DT-C, LC-GAD, and PedsQL FIM-A. Some instruments, such as the COPE Index and Brief COPE, showed low-quality evidence for internal consistency. This prevalence of low-quality evidence indicates a need for more rigorous evaluation of these properties across most instruments.

Very low--quality evidence was predominantly found for cross-cultural validity. ZBI, BDI, HD-DT-C, COPE Index, SPBS, ZCBS, ZBI-A, Brief COPE, and PedsQL FIM-A all showed very low--quality evidence for cross-cultural validity. ZBI-A also demonstrated very low--quality evidence for internal consistency. This consistent pattern suggests a critical gap in the evaluation of cross-cultural validity for caregiver burden instruments in ESKD.

For many instruments, evidence was entirely lacking for one or more measurement properties. This was particularly true for measurement errors, criterion validity, and responsiveness, for which most instruments had no available data. This absence of evidence highlights critical gaps in the current research. This suggests a systemic gap in the evaluation of these properties in caregiver burden instruments for ESKD.

### Patient and Public Involvement

Patients and public were not involved in the design or conduct of this study. Because this report is a protocol outlining plans for a systematic review, there was no opportunity for patient participation in data collection or intervention activities.

## Discussion

This systematic review comprehensively evaluated the measurement properties of caregiver burden instruments used in ESKD against the COSMIN criteria. To our knowledge, this is the first systematic review to critically appraise the measurement properties of caregiver burden instruments and grade the quality of evidence in ESKD using the rigorous COSMIN approach.

We identified 21 caregiver burden instruments applied in ESKD, highlighting the diversity of measures used in this population. However, for most instruments, evidence on one or more measurement properties was lacking or of low to very low quality. No instrument had high-quality evidence of sufficient validity, reliability, and responsiveness.

ZBI, CBS, BDI, and PR-CBS were among the most frequently evaluated instruments and demonstrated moderate-quality evidence of sufficient internal consistency and reliability. This suggests that the item scores of these instruments are interrelated, and they are likely to measure the intended construct of caregiver burden consistently. However, even for these commonly used instruments, evidence was of low or very low quality for other important properties such as cross-cultural validity and hypothesis testing. This limits confidence in the cross-cultural applicability and construct validity of these instruments.

Furthermore, many of the evaluated instruments (eg, ZBI, CBS, BDI, COPE Index, SPBS, ZCBS, and Brief COPE) were originally developed for caregivers of non-ESKD populations and may not fully capture the unique aspects of burden experienced by caregivers of patients with kidney failure. Content validity studies involving caregivers of patients with kidney failure are essential to ensure the relevance, comprehensiveness, and comprehensibility of these instruments. Only a few ESKD-specific instruments (HD-DT-C, PR-CBS, PCTQHFC, HD-PCBS, LC-GAD, CBQ, and PedsQL FIM-A) have been developed, but these had limited evidence of their measurement properties. Further validation of these promising instruments is required before they can be recommended for routine use. The structural validity of many instruments (eg, COPE Index, PCTQHFC, HD-PCBS, LC-GAD, and OQCPPD) was supported by low-quality evidence, indicating a need for more robust factor analysis studies with larger sample sizes. Hypothesis testing, which is crucial for establishing construct validity, was also often supported by low-quality evidence or not reported at all for many instruments. This limits our confidence in the instruments’ ability to accurately measure the intended construct of caregiver burden in ESKD.

It is worth noting that some newer or less-common instruments (eg, PCTQHFC, OQCPPD, and CBQ) had very limited reported evidence across most properties. This highlights the need for comprehensive evaluation of these tools before they can be considered for widespread use in ESKD caregiver research or clinical practice.

Strikingly, no studies evaluated the responsiveness of instruments to change in caregiver burden over time, a key requirement for evaluative applications such as testing the effectiveness of caregiver interventions.^16^^,^^17^ This gap may reflect the predominance of cross-sectional designs, small samples, and the lack of successful interventions for ESKD caregiver burden to date.^6^^,^^13^ Longitudinal studies with larger samples powered to detect change are needed.

When comparing the psychometric evidence for caregiver burden instruments in ESKD with those used in other chronic life-limiting conditions such as heart failure, cancer, and dementia, several patterns emerge. Caregiver burden instruments in these other conditions typically demonstrate more extensive validation, particularly for responsiveness to change.^47^^,^^48^ For example, ZBI has established minimal important difference values and demonstrated responsiveness in dementia caregiving contexts^49^ but lacks such evidence in ESKD. Similarly, cancer-specific instruments such as the Caregiver Reaction Assessment show more robust evidence for cross-cultural validity across diverse populations than ESKD instruments.^50^^,^^51^ Heart failure caregiver burden measures often include disease-specific modules that have undergone systematic content validation with both caregivers and clinicians,^52^^,^^53^ an approach less common in ESKD. The measurement gap is particularly notable for responsiveness, in which instruments in dementia (eg, ZBI) and cancer (eg, Caregiver Quality of Life Index-Cancer) have established sensitivity to change in intervention studies,^54^^,^^55^ whereas no ESKD instruments have such validation. These comparisons highlight opportunities for the ESKD field to adopt methodological advances from other conditions, particularly in developing responsive measures suitable for intervention trials and establishing clinically meaningful change thresholds.

We also found a paucity of studies on cross-cultural validity and measurement invariance. Most instruments were developed in English in western countries and may not perform equivalently in other languages and cultures without adaptation.^56^^,^^57^ For western countries, ZBI and CBS currently represent the most reasonable options for clinical and research applications based on our findings. Both instruments have demonstrated moderate-quality evidence for internal consistency, reliability, and structural validity in western populations. ZBI, with its established cutoff scores and widespread use, facilitates interpretation and comparison across studies. For settings requiring briefer assessment, ZBI-12 offers a valid alternative with comparable psychometric properties. CBS provides more granular assessment through its multidimensional structure capturing distinct aspects of burden (general strain, isolation, disappointment, emotional involvement, and environment). For specialized contexts, HD-DT-C shows promise as an ESKD-specific screening tool developed in a western context, although it requires further validation beyond its initial development. These instruments should be selected with consideration of the specific research or clinical question, burden domains of interest, and practical constraints such as administration time. As the global prevalence of treated ESKD increases,^2^^,^^3^ there is an urgent need to cross-culturally validate instruments to enable appropriate assessment and comparisons of caregiver burden across diverse populations.

This review makes several novel contributions to the field. It is the first to apply the latest COSMIN guidelines to systematically evaluate the measurement properties of caregiver burden instruments in ESKD, providing a rigorous and transparent quality assessment using the Grading of Recommendations Assessment, Development and Evaluation approach.^16^^,^^21^ Prior reviews have described burden instruments used in ESKD^13^^,^^14^ but have not critically appraised their measurement properties against established standards. By doing so, this review provides a more nuanced understanding of the strengths and limitations of current measures and the overall state of validation evidence. The findings show significant limitations in existing research and identify key evidence gaps to guide future validation studies. Importantly, the review highlights the low methodological quality of many studies as a crucial issue that needs to be addressed to advance the science of caregiver burden measurement in ESKD. Rather than invalidating the results, this finding underscores the need for more rigorous research practices and provides a roadmap for improving future studies.

### Strengths and Limitations

A key strength of our review is the comprehensive search strategy and rigorous screening process following the Preferred Reporting Items for Systematic Reviews and Meta-analyses guidelines. By including studies of varying methodological quality and transparently reporting evidence quality, this review provides a complete and unbiased picture of the current state of research.

Another strength of our review is the use of the COSMIN methodology, the current gold standard for evaluating patient-reported outcome measures.^16^^,^^21^ We used the latest COSMIN tools to assess risk of bias (methodological quality) and applied the updated criteria for good measurement properties. This enabled standardized evaluation and comparison across different instruments and studies. Furthermore, we graded the quality of the evidence for each measurement property of each instrument using the Grading of Recommendations Assessment, Development and Evaluation approach adapted for this purpose.^16^^,^^21^ This provided a transparent assessment of the certainty of the evidence.

However, our review has some limitations. We only included English language articles, potentially excluding relevant studies. Owing to the large variability in study characteristics and instrument properties, a meta-analysis was not possible. The inconsistent reporting of study methods and results also hampered comparability and synthesis. Finally, no studies evaluated measurement errors, criterion validity, or responsiveness, limiting conclusions about these important properties.

### Implications for Practice and Research

Researchers and clinicians should exercise caution when selecting caregiver burden instruments for ESKD, considering the limited high-quality evidence available. ZBI, CBS, and BDI show promise but require further validation.^13^ Priority should be given to content validity studies involving caregivers of patients with kidney failure,^58^ rigorous evaluations of all measurement properties, and cross-cultural validation.^56^ Larger, longitudinal studies are needed to assess responsiveness.^59^ Implementation in clinical practice requires training, electronic systems for data capture, and evidence-based interventions linked to assessment results.^38^

Despite the limitations of current evidence, this review provides valuable information for clinicians and researchers. Understanding the strengths and weaknesses of available instruments, even if not perfect, can inform more nuanced selection and interpretation. From a clinical perspective, this nuanced understanding can significantly improve assessment practices and patient family care. Clinicians could select instruments based on their specific measurement properties aligned with clinical goals—for example, using instruments with strong content validity and internal consistency (such as CBS or ZBI) for initial burden screening, while recognizing their limitations for measuring change over time because of unestablished responsiveness. When implementing these instruments in dialysis centers, nephrology practices, or palliative care settings, clinicians should consider the multidimensional nature of burden; instruments with established factor structures (eg, CBS with its 5 dimensions) can help identify specific domains requiring intervention, such as social isolation or emotional strain. Interpretation of scores should account for the quality of evidence supporting cutoff values—for instance, although ZBI cutoffs are widely used, the evidence quality supporting these thresholds specifically in ESKD is limited. Clinicians might also consider supplementing standardized assessments with qualitative exploration of burden experiences, particularly in culturally diverse populations in which cross-cultural validity evidence is limited. This approach allows for more personalized care planning, targeted interventions addressing specific burden dimensions, and more accurate monitoring of caregiver wellbeing over the ESKD trajectory, ultimately supporting sustainable caregiving arrangements that benefit both caregivers and patients. The findings also underscore the importance of considering evidence quality when choosing or evaluating measures and the need for ongoing validation research. Excluding low-quality studies would paint an incomplete picture and risk overconfidence in inadequately evaluated instruments.

Caregiver involvement in research and instrument development is crucial.^60^ A shift in policy is needed to recognize and support caregivers of patients with kidney failure, integrating caregiver outcomes into clinical guidelines and reimbursement models.^61^^,^^62^

### Future Research Directions

There is a critical need for high-quality studies evaluating the measurement properties of caregiver burden instruments in ESKD. Cross-cultural validity, measurement errors, criterion validity, and responsiveness require particular attention, because it consistently showed very low-quality evidence or lack of evidence across most instruments. Newer or less-common instruments (eg, PCTQHFC, OQCPPD, and CBQ) need more comprehensive evaluation across all measurement properties. Even well-established instruments such as ZBI would benefit from further high-quality studies to strengthen their evidence base.

Key areas for future research include developing and validating ESKD-specific caregiver burden instruments; conducting longitudinal studies to understand burden trajectories and instrument responsiveness; performing cross-cultural validation studies^57^; evaluating measurement error and minimal important difference^21^; comparing different instruments head –to head; investigating the implementation of caregiver burden assessment in clinical practice^63^; developing and testing interventions based on burden assessments^14^; exploring the relationship between caregiver burden and patient outcomes^9^; examining burden across different ESKD treatment modalities; and using modern psychometric methods such as item response theory and computer adaptive testing.^64^ These efforts will contribute to a more robust evidence base for caregiver burden assessment in ESKD, ultimately improving outcomes for both caregivers and patients.^11^

Such studies should incorporate several key characteristics to substantially advance the field, which are as follows: (1) adequate sample sizes with clear reporting of caregiver demographics, relationship with the patient, and caregiving duration; (2) comprehensive conceptual framework development through qualitative research with diverse caregivers of patients with kidney failure to ensure content validity across different treatment modalities; (3) robust psychometric methodology including CFA, measurement invariance testing across key subgroups (eg, spousal vs nonspousal caregivers), and modern psychometric approaches such as item response theory; (4) longitudinal designs with multiple assessment points to establish responsiveness to change and minimal important difference values; (5) multicenter, cross-cultural designs with proper adaptation procedures to enhance generalizability; and (6) direct comparison with existing instruments to establish convergent validity and relative measurement performance. These methodological improvements would substantially strengthen the evidence base for caregiver burden assessment in ESKD.

## Conclusion

This study applied rigorous COSMIN methodology to identify and critically appraise instruments for assessing subjective caregiver burden in ESKD. Our findings show significant limitations in research assessing the measurement properties of these instruments and low to very low evidence for their validity, reliability, and responsiveness. ZBI, CBS, and BDI were most widely used and currently have evidence of internal consistency and construct validity but require further testing of other properties. Key research priorities include establishing content validity with caregivers of patients with kidney failure, evaluating responsiveness, and cross-cultural validation. Although the low methodological quality of many studies poses challenges for drawing definitive conclusions, this transparency about evidence limitations is a strength, not a weakness, of the review. It highlights the need for more rigorous validation research in this field. Researchers, clinicians, and policymakers must consider the quality of evidence when selecting and interpreting caregiver burden measures. A collaborative, caregiver-engaged approach to instrument development, validation, implementation, and policymaking is recommended to optimize the relevance and effectiveness of burden assessment in ESKD. Ultimately, high-quality measures are necessary, but not sufficient, to improve the lives of caregivers who play a critical role in supporting patients with ESKD.

The findings of this review underscore the need for continued research and development in the field of caregiver burden assessment in ESKD. As the global burden of ESKD continues to increase, the role of caregivers becomes increasingly critical. Accurate and comprehensive assessment of caregiver burden is essential for identifying those at risk, developing targeted interventions, and evaluating the effectiveness of support strategies. By addressing the gaps identified in this review and pursuing the suggested research directions, we can work toward a more robust evidence base for caregiver burden assessment in ESKD, ultimately leading to improved outcomes for both caregivers and patients. It is our hope that this review will serve as a catalyst for high-quality research in this important area.

## Supplementary Materials

Supplementary File (PDF) Items S1-S4.
