# Supplementary material for: Assessing Caregiver Burden in Kidney Failure: A Systematic Review of Measurement Properties of Instruments
Source: Kidney Med. 2025 Jun 24;7(9):101054. doi: 10.1016/j.xkme.2025.101054 (PMC12337670; doi:10.1016/j.xkme.2025.101054)
Supplement: Supplementary File (PDF) — Items S1-S4. [file mmc1.pdf]

Item S1: PRISMA 2020 Checklist

Section and Topic	Item #	Checklist item	Location where item is reported
**TITLE**			
Title	1	Identify the report as a systematic review.	Title page
**ABSTRACT**			
Abstract	2	See the PRISMA 2020 for Abstracts checklist.	Abstract section
**INTRODUCTION**			
Rationale	3	Describe the rationale for the review in the context of existing knowledge.	Introduction, paragraphs 1-5
Objectives	4	Provide an explicit statement of the objective(s) or question(s) the review addresses.	Introduction, last paragraph
**METHODS**			
Eligibility criteria	5	Specify the inclusion and exclusion criteria for the review and how studies were grouped for the syntheses.	Methods: 2.1 Eligibility criteria, Table 1
Information sources	6	Specify all databases, registers, websites, organisations, reference lists and other sources searched or consulted to identify studies. Specify the date when each source was last searched or consulted.	Methods: 2.2 Search strategy
Search strategy	7	Present the full search strategies for all databases, registers and websites, including any filters and limits used.	Methods: 2.2 Search strategy
Selection process	8	Specify the methods used to decide whether a study met the inclusion criteria of the review, including how many reviewers screened each record and each report retrieved, whether they worked independently, and if applicable, details of automation tools used in the process.	Methods: 2.3 Study selection
Data collection process	9	Specify the methods used to collect data from reports, including how many reviewers collected data from each report, whether they worked independently, any processes for obtaining or confirming data from study investigators, and if applicable, details of automation tools used in the process.	Methods: 2.4 Data extraction
Data items	10a	List and define all outcomes for which data were sought. Specify whether all results that were compatible with each outcome domain in each study were sought (e.g. for all measures, time points, analyses), and if not, the methods used to decide which results to collect.	Methods: 2.4 Data extraction, Appendix II
	10b	List and define all other variables for which data were sought (e.g. participant and intervention characteristics, funding sources). Describe any assumptions made about any missing or unclear information.	Methods: 2.4 Data extraction, Appendix II
Study risk of bias assessment	11	Specify the methods used to assess risk of bias in the included studies, including details of the tool(s) used, how many reviewers assessed each study and whether they worked independently, and if applicable, details of automation tools used in the process.	Methods: 2.5 Methodological quality assessment
Effect measures	12	Specify for each outcome the effect measure(s) (e.g. risk ratio, mean difference) used in the synthesis or presentation of results.	Not applicable (qualitative synthesis)

Section and Topic	Item #	Checklist item	Location where item is reported
Synthesis methods	13a	Describe the processes used to decide which studies were eligible for each synthesis (e.g. tabulating the study intervention characteristics and comparing against the planned groups for each synthesis (item #5)).	Methods: 2.6 Data Synthesis and Quality Assessment
	13b	Describe any methods required to prepare the data for presentation or synthesis, such as handling of missing summary statistics, or data conversions.	Methods: 2.6 Data Synthesis and Quality Assessment
	13c	Describe any methods used to tabulate or visually display results of individual studies and syntheses.	Methods: 2.6 Data Synthesis and Quality Assessment, Tables 2-8
	13d	Describe any methods used to synthesize results and provide a rationale for the choice(s). If meta-analysis was performed, describe the model(s), method(s) to identify the presence and extent of statistical heterogeneity, and software package(s) used.	Methods: 2.6 Data Synthesis and Quality Assessment
	13e	Describe any methods used to explore possible causes of heterogeneity among study results (e.g. subgroup analysis, meta-regression).	Not applicable (qualitative synthesis)
	13f	Describe any sensitivity analyses conducted to assess robustness of the synthesized results.	Not applicable
Reporting bias assessment	14	Describe any methods used to assess risk of bias due to missing results in a synthesis (arising from reporting biases).	Not reported
Certainty assessment	15	Describe any methods used to assess certainty (or confidence) in the body of evidence for an outcome.	Methods: 2.6 Data Synthesis and Quality Assessment, Table 3
**RESULTS**			
Study selection	16a	Describe the results of the search and selection process, from the number of records identified in the search to the number of studies included in the review, ideally using a flow diagram.	Results: 3.1 Search results, Figure 2
	16b	Cite studies that might appear to meet the inclusion criteria, but which were excluded, and explain why they were excluded.	Results: 3.1 Search results
Study characteristics	17	Cite each included study and present its characteristics.	Results: 3.2 Study characteristics, Tables 4-6
Risk of bias in studies	18	Present assessments of risk of bias for each included study.	Results: 3.5 Overall quality of evidence, Table 8
Results of individual studies	19	For all outcomes, present, for each study: (a) summary statistics for each group (where appropriate) and (b) an effect estimate and its precision (e.g. confidence/credible interval), ideally using structured tables or plots.	Results: 3.4 Results on measurement properties, Tables 7-8

Section and Topic	Item #	Checklist item	Location where item is reported
Results of syntheses	20a	For each synthesis, briefly summarise the characteristics and risk of bias among contributing studies.	Results: 3.5 Overall quality of evidence
	20b	Present results of all statistical syntheses conducted. If meta-analysis was done, present for each the summary estimate and its precision (e.g. confidence/credible interval) and measures of statistical heterogeneity. If comparing groups, describe the direction of the effect.	Not applicable (qualitative synthesis)
	20c	Present results of all investigations of possible causes of heterogeneity among study results.	Not applicable
	20d	Present results of all sensitivity analyses conducted to assess the robustness of the synthesized results.	Not applicable
Reporting biases	21	Present assessments of risk of bias due to missing results (arising from reporting biases) for each synthesis assessed.	Not reported
Certainty of evidence	22	Present assessments of certainty (or confidence) in the body of evidence for each outcome assessed.	Results: 3.5 Overall quality of evidence, Table 8
**DISCUSSION**			
Discussion	23a	Provide a general interpretation of the results in the context of other evidence.	Discussion, paragraphs 1-5
	23b	Discuss any limitations of the evidence included in the review.	Discussion: 4.1 Strengths and limitations
	23c	Discuss any limitations of the review processes used.	Discussion: 4.1 Strengths and limitations
	23d	Discuss implications of the results for practice, policy, and future research.	Discussion: 4.2 Implications for practice and research, 4.3 Future research directions
**OTHER INFORMATION**			
Registration and protocol	24a	Provide registration information for the review, including register name and registration number, or state that the review was not registered.	Methods: The review protocol was registered in PROSPERO (registration number: CRD42023433906)
	24b	Indicate where the review protocol can be accessed, or state that a protocol was not prepared.	Not reported
	24c	Describe and explain any amendments to information provided at registration or in the protocol.	Not reported

Section and Topic	Item #	Checklist item	Location where item is reported
Support	25	Describe sources of financial or non-financial support for the review, and the role of the funders or sponsors in the review.	Funding section
Competing interests	26	Declare any competing interests of review authors.	Competing Interests section
Availability of data, code and other materials	27	Report which of the following are publicly available and where they can be found: template data collection forms; data extracted from included studies; data used for all analyses; analytic code; any other materials used in the review.	Not reported

From: Page MJ, McKenzie JE, Bossuyt PM, Boutron I, Hoffmann TC, Mulrow CD, et al. The PRISMA 2020 statement: an updated guideline for reporting systematic reviews. BMJ 2021;372:n71. doi: 10.1136/bmj.n71

Item S2: Search strategies for different databases

Search strategy for PubMed

Steps	Search String
1	(("caregiv*"[tiab] OR "caregiv*"[mh]) and ("burden" [tiab] OR " stress*"[tiab] OR "strain*"[tiab] OR "distress*"[tiab] OR "load"[tiab] OR "fatigue"[tiab] OR "burnout"[tiab] OR "exhaust*"[tiab] OR "overwhelm*"[tiab] OR "anxiety"[tiab] OR "depressi*"[tiab] OR "emotional strain*"[tiab] OR "psychological distress*"[tiab] OR "emotional burden*"[tiab] OR "physical burden*"[tiab] OR "mental burden*"[tiab] OR "hardship*"[tiab] OR "challeng*"[tiab] OR "difficult*"[tiab] OR "demand*"[tiab] OR "responsibilit*"[tiab] OR "impact*"[tiab] OR "pressur*"[tiab] OR "demand*"[tiab] OR "stressors"[tiab])) AND ((humans[filter]) AND (1960/1/1:2023/06/15[pdat]) AND (english[filter]) AND (alladult[filter]))
2	(("advanced kidney*"[tiab] OR "chronic kidney disease stage 5"[tiab] OR "stage 5 chronic kidney disease"[tiab] "CKD stage 5"[tiab] OR "stage 5 CKD"[tiab] OR "CKD 5"[tiab] OR "CKD-5"[tiab] OR "end-stage renal disease"[tiab] OR "end-stage kidney disease"[tiab] OR "renal failure stage 5"[tiab] OR "renal insufficiency stage 5"[tiab] OR "kidney dysfunction stage 5"[tiab] OR "ESRD"[tiab] OR "dialysis” OR "dialysis-dependent patients"[tiab] OR "Hemodialysis"[tiab] OR "Haemodialysis"[tiab] OR "Peritoneal Dialysis"[tiab] OR "Continuous Ambulatory Peritoneal Dialysis"[tiab] OR "Automated Peritoneal Dialysis"[tiab] OR "Hemofiltration"[tiab] OR "Hemodiafiltration"[tiab] OR "Hemoperfusion"[tiab] OR "Slow Continuous Ultrafiltration"[tiab] OR "Continuous Renal Replacement Therapy"[tiab] OR "Sustained Low-Efficiency Dialysis"[tiab] OR "Nocturnal Dialysis"[tiab] OR "Short Daily Home Hemodialysis"[tiab] OR "Short Daily Home Haemodialysis"[tiab] OR "Home Hemodialysis"[tiab] OR "Home Haemodialysis"[tiab] OR "HD"[tiab] OR "PD"[tiab] OR "CAPD"[tiab] OR "APD"[tiab] OR "HF"[tiab] OR "HDF"[tiab] OR "HP"[tiab] OR "SCUF"[tiab] OR "CRRT"[tiab] OR "SLED"[tiab] OR "ND"[tiab] OR "SDD"[tiab] OR "HHD"[tiab] OR "Hemodialysis"[mh] OR "Haemodialysis"[mh] OR "Peritoneal Dialysis"[mh] OR "Continuous Ambulatory Peritoneal Dialysis"[mh] OR "Automated Peritoneal Dialysis"[mh] OR "Hemofiltration"[mh] OR "Hemodiafiltration"[mh] OR "Hemoperfusion"[mh] OR "Slow Continuous Ultrafiltration"[mh] OR "Continuous Renal Replacement Therapy"[mh] OR "Sustained Low-Efficiency Dialysis"[mh] OR "Nocturnal Dialysis"[mh] OR "Short Daily Home Hemodialysis"[mh] OR "Short Daily Home Haemodialysis"[mh] OR "Home Hemodialysis"[mh] OR "Home Haemodialysis"[mh] OR "HD"[mh] OR "PD"[mh] OR "CAPD"[mh] OR "APD"[mh] OR "HF"[mh] OR "HDF"[mh] OR "HP"[mh] OR "SCUF"[mh] OR "CRRT"[mh] OR "SLED"[mh] OR "ND"[mh] OR "SDD"[mh] OR "HHD"[mh] OR "renal transplant recipients"[tiab] OR "transplant"[tiab] OR "nephrology patients"[title/abstract]) OR ("patients with*"[tiab] AND "severe kidney disease"[tiab] OR “advanced renal impairment” [tiab] OR " advanced kidney dysfunction"[tiab] OR "severe renal insufficiency"[tiab] OR "significant decline in kidney function"[tiab] OR "critically impaired kidney function"[tiab] OR "end-stage renal dysfunction"[tiab] OR "profound renal compromise"[tiab] OR "significant kidney damage"[tiab] OR "advanced kidney pathology"[tiab] OR "late-stage kidney disease"[tiab] OR "advanced renal failure"[tiab] OR "severe renal disorder"[tiab] OR "advanced renal dysfunction"[tiab] OR "critical kidney illness"[tiab] OR "profound kidney impairment"[tiab] OR "advanced kidney pathology"[tiab] OR "late-stage renal disease"[tiab] OR "advanced renal insufficiency"[tiab])) AND ((humans[filter]) AND (1960/1/1:2023/06/08[pdat]) AND (english[filter]) AND (alladult[filter]))

3	("assessment tools"[tiab] OR "evaluation instruments"[tiab] OR "rating scales"[tiab] OR "surveys"[tiab] OR "inventories"[tiab] OR "questionnaires"[tiab] OR "checklists"[tiab] OR "measur*"[tiab] OR "tests"[tiab] OR "protocols"[tiab] OR "indexes"[tiab] OR "tools"[tiab] OR "instruments of measurement"[tiab] OR "diagnostic tools"[tiab] OR "observational tools"[tiab] OR "screening tools"[tiab] OR "psychometric instruments"[tiab] OR "psychosocial measurement tools"[tiab] OR ("caregiver burden"[tiab] and “scales” OR "questionnaires"[tiab] OR "inventories"[tiab] OR "assessments"[tiab] OR "surveys"[tiab] OR "measures"[tiab] OR "instruments"[title/abstract]) OR "caregiver strain tools"[tiab] OR "caregiver stress measures"[tiab] OR "caregiver distress scales"[tiab] OR "caregiver impact questionnaires"[tiab] OR "caregiver load inventories"[tiab] OR "caregiver challenges assessments"[tiab] OR "caregiver difficulties scales"[tiab] OR "caregiver well-being measures"[tiab] OR "Zarit Burden Interview"[tiab] OR "SF-36"[tiab] OR "CES-D"[tiab] OR "BDI"[tiab] OR "DAS"[tiab] OR "HADS"[tiab] OR "PSQI"[tiab] OR "CBS"[tiab] OR "BSI"[tiab] OR "Psychological Adjustment to Illness Scale"[tiab] OR "Jaloweic Coping Scale"[tiab] OR "Caregiver Burden Inventory"[tiab] OR "Caregiver Strain Index"[tiab]) AND ((humans[filter]) AND (1960/1/1:2023/06/15[pdat]) AND (english[filter]) AND (alladult[filter]))
4	("test-retest reliability"[mh] OR "test-retest reliability"[tiab] OR "inter-rater reliability"[mh] OR "inter-rater reliability"[tiab] OR "intra-rater reliability"[mh] OR "intra-rater reliability"[tiab] OR "convergent validity"[mh] OR "convergent validity"[tiab] OR "discriminant validity"[mh] OR "discriminant validity"[tiab] OR "criterion validity"[mh] OR "criterion validity"[tiab] OR "factor analysisl"[mh] OR "factOR analysis "[tiab] OR "principal component analysis"[mh] OR "principal component analysis"[tiab] OR "item analysis"[mh] OR "item analysis"[tiab] OR "item response theory"[mh] OR "item response theory"[tiab] OR "rasch analysis"[mh] OR "rasch analysis"[tiab] OR "differential item functioning"[mh] OR "differential item functioning"[tiab] OR "reliability and validity"[mh] OR "reliability and validity"[tiab] OR instrumentation[sh] OR methods[sh] OR Validation Studies[pt] OR Comparative Study[pt] OR ‘‘psychometrics’’ [mh] OR psychometr*[tiab] OR clinimetr*[tw] OR clinometr*[tw] OR ‘‘outcome assessment (health care)’’[mh] OR outcome assessment[tiab] OR outcome measure*[tw] OR ‘‘observer variation’’[mh] OR observer variation[tiab] OR ‘‘Health Status Indicators’’[mh] OR ‘‘reproducibility of results’’[mh] OR reproducib*[tiab] OR ‘‘discriminant analysis’’[mh] OR reliab*[tiab] OR unreliab*[tiab] OR valid*[tiab] OR coefficient[tiab] OR homogeneity[tiab] OR homogeneous[tiab] OR ‘‘internal consistency’’[tiab] OR (cronbach*[tiab] AND (alpha[tiab] OR alphas[tiab])) OR (item[tiab] AND (correlation*[tiab] OR selection*[tiab] OR reduction*[tiab])) OR agreement[tiab] OR precision[tiab] OR imprecision[tiab] OR ‘‘precise values’’[tiab] OR test– retest[tiab] OR (test[tiab] AND retest[tiab]) OR (reliab* [tiab] AND (test[tiab] OR retest[tiab])) OR stability[tiab] OR interrater[tiab] OR inter-rater[tiab] OR intrarater[tiab] OR intra-rater[tiab] OR intertester[tiab] OR inter-tester[tiab] OR intratester[tiab] OR intra-tester[tiab] OR interobserver[tiab] OR inter-observer[tiab] OR intraobserver[tiab] OR intraobserver[tiab] OR intertechnician[tiab] OR inter-technician[tiab] OR intratechnician[tiab] OR intra-technician[tiab] OR interexaminer[tiab] OR inter-examiner[tiab] OR intraexaminer[tiab] OR intra-examiner[tiab] OR interassay[tiab] OR inter-assay[tiab] OR intraassay[tiab] OR intra-assay[tiab] OR interindividual[tiab] OR inter-individual[tiab] OR intraindividual[tiab] OR intra-individual[tiab] OR interparticipant [tiab] OR inter-participant[tiab] OR intraparticipant[tiab] OR intra-participant[tiab] OR kappa[tiab] OR kappa’s[tiab] OR kappas[tiab] OR repeatab*[tiab] OR ((replicab*[tiab] OR repeated[tiab]) AND (measure[tiab] OR measures[tiab] OR findings[tiab] OR result[tiab] OR results[tiab] OR test[- tiab] OR tests[tiab])) OR generaliza*[tiab] OR generalisa*[tiab] OR concordance[tiab] OR (intraclass[tiab] AND correlation*[tiab]) OR discriminative[tiab] OR ‘‘known group’’[tiab] OR factor analysis[tiab] OR factor analyses[tiab] OR dimension*[tiab] OR subscale*[tiab] OR (multitrait[tiab] AND scaling[tiab] AND (analysis[tiab] OR analyses[tiab])) OR item discriminant[tiab] OR interscale correlation*[tiab] OR error[tiab] OR errors[tiab] OR

	‘‘individual variability’’[tiab] OR (variability[tiab] AND (analysis[tiab] OR values[tiab])) OR (uncertainty[tiab] AND (measurement[tiab] OR measuring[tiab])) OR ‘‘standard error of measurement’’[tiab] OR sensitiv*[tiab] OR responsive*[tiab] OR ((minimal[tiab] OR minimally[tiab] OR clinical[tiab] OR clinically[tiab]) AND (important[tiab] OR significant[tiab] ORdetectable[tiab])AND(change[tiab]ORdifference[tiab])) OR (small*[tiab] AND (real[tiab] OR detectable[tiab]) AND (change[tiab] OR difference[tiab])) OR meaningful change [tiab] OR ‘‘ceiling effect’’[tiab] OR ‘‘floor effect’’[tiab] OR ‘‘Item response model’’[tiab] OR IRT[tiab] OR Rasch[tiab] OR ‘‘Differential item functioning’’[tiab] OR DIF[tiab] OR ‘‘computer adaptive testing’’[tiab] OR ‘‘item bank’’[tiab] OR ‘‘cross-cultural equivalence’’[tiab]) AND ((humans[filter]) AND (1960/1/1:2023/06/15[pdat]) AND (english[filter]) AND (alladult[filter]))
5	#1 AND #2 AND #3 AND #4
6	("qualitative study"[pt] OR "case reports"[pt] OR "editorial"[pt] OR "legal cases"[pt] OR "Delphi technique"[tiab] OR "cross-sectional"[tiab] OR ‘‘addresses’’[pt] OR ‘‘biography’’[pt] OR "lectures"[pt] OR "newspaper article"[pt] OR "review"[pt] OR "letter"[pt] OR "news"[pt] OR "interview"[pt] OR "comment"[pt] OR "guideline"[pt] OR "consensus development conference"[pt] OR "practice guideline"[pt] OR "opinion"[pt] OR "book chapters"[pt] OR "letter"[pt] OR "conference abstract"[pt] OR ‘‘directory’’[pt] OR ‘‘festschrift’’[pt] OR ‘‘legislation’’[pt] OR ‘‘patient education handout’’[pt] OR ‘‘popular works’’[pt] OR ‘‘congresses’’ [pt] OR ‘‘practice guideline’’[pt] OR "in vitro techniques"[mh] OR "laboratory"[mh] OR "cell culture"[mh] OR "pediatrics"[mh] OR "early-stage kidney disease"[tiab]) AND ((humans[filter]) AND (1960/1/1:2023/06/15[pdat]) AND (english[filter]) AND (alladult[filter]))
7	#5 NOT #6

Search string for CINAHL, Embase, MEDLINE, PsycINFO, Cochrane Library, SCOPUS, and Web of Science databases

(('caregiver' OR 'caregiving') AND ('burden' OR 'stress' OR 'strain' OR 'distress' OR 'load' OR 'fatigue' OR 'burnout' OR 'exhaustion' OR 'overwhelm' OR 'anxiety' OR 'depression' OR 'emotional AND strain' OR 'psychological AND distress' OR 'emotional AND burden' OR 'physical AND burden' OR 'mental AND burden' OR 'hardship' OR 'challenge' OR 'difficulty' OR 'demand' OR 'responsibility' OR 'impact' OR 'pressure' OR 'stressors')) AND ('kidney AND disease' OR 'advanced AND kidney AND disease' OR 'haemodialysis' OR 'dialysis' OR 'peritoneal AND dialysis')

Constructs and related terms

Caregiver burden:

Caregiver burden; Caregiver stress; Caregiver strain; Caregiver distress; Caregiver load; Caregiver fatigue; Caregiver burnout; Caregiver exhaustion; Caregiver overwhelm; Caregiver anxiety; Caregiver depression; Caregiver emotional strain; Caregiver psychological distress; Caregiver emotional burden; Caregiver physical burden; Caregiver mental burden; Caregiver hardship; Caregiver challenges; Caregiver difficulties; Caregiver demands; Caregiver responsibilities; Caregiver impact; Caregiver strain and stress; Caregiving pressure; Caregiving strain; Caregiving demands; Caregiving challenges; Caregiving difficulties; Caregiving stressors; Care recipient burden

Advanced kidney disease:

Kidney Failure; Chronic Kidney Disease; Kidney Disease;; Renal Insufficiency; Renal Replacement Therapy; Hemodialysis; Peritoneal Dialysis; Kidney Transplantation; End stage renal disease; End stage kidney disease; Renal failure stage 5; Dialysis dependent; Hemodialysis patient*; Peritoneal dialysis patient*; Renal transplant recipient*; Nephrology patient*; Severe kidney disease; Advanced renal impairment; Advanced kidney dysfunction; Severe renal insufficiency; Significant decline in kidney function; Critically impaired kidney function; End-stage renal dysfunction; Profound renal compromise; Late-stage kidney disease; Advanced renal failure; Severe renal disorder; Critical kidney illness; Profound kidney impairment; Late-stage renal disease; Advanced renal insufficiency; Severe renal dysfunction; Critically impaired renal function; Significant renal damage; Late-stage kidney dysfunction; Advanced kidney failure; Critical renal illness; Late-stage renal insufficiency; Late-stage renal failure; Significant decline in renal function; End-stage kidney dysfunction; Advanced kidney dysfunction; Severe kidney dysfunction; Critically impaired kidney function; Late-stage kidney disease; Severe kidney disease; Critical kidney dysfunction; Late-stage renal dysfunction; Advanced kidney insufficiency; Profound kidney insufficiency; Critical renal dysfunction; Late-stage renal failure; Severe renal insufficiency; Late-stage renal disease; Significant kidney damage; Late-stage kidney failure

Instruments:

Assessment tools; Evaluation instruments; Rating scales; Surveys; Inventories; Questionnaires; Checklists; Measures; Tests; Protocols; Indexes; Instruments of measurement; Diagnostic tools; Observational tools; Screening tools; Psychometric instruments; Psychosocial measurement tools

Caregiver Burden Measurement Tools:

Caregiver burden scales; Caregiver burden questionnaires; Caregiver burden inventories; Caregiver burden assessments; Caregiver burden surveys; Caregiver burden measures; Caregiver burden instruments; Caregiver strain tools; Caregiver stress measures; Caregiver distress scales; Caregiver impact questionnaires; Caregiver load inventories; Caregiver challenges assessments; Caregiver difficulties scales; Caregiver well-being measures

Specific Measurement Tools:

Zarit Burden Interview (ZBI), SF-36 (Short Form 36), CES-D (Centre for Epidemiological Studies Depression Scale), BDI (Beck Depression Inventory), DAS (Depression Anxiety and Stress Scale), HADS (Hospital Anxiety and Depression Scale), PSQI (Pittsburgh Sleep Quality Index), CBS (Caregiver Burden Scale), BSI (Brief Symptom Inventory), Psychological Adjustment to Illness Scale (PAIS), Jaloweic Coping Scale (JCS), Caregiver Burden Inventory (CBI), Caregiver Strain Index (CSI)

Psychometric Properties:

Reliability; Validity; Sensitivity; Specificity; Responsiveness; Feasibility; Internal consistency; Test-retest reliability; Inter-rater reliability; Intra-rater reliability; Concurrent validity; Predictive validity; Construct validity; Convergent validity; Discriminant validity; Criterion validity; Factor analysis; Factor structure; Item analysis; Floor effect; Ceiling effect; Content validity; Face validity; Concurrent validity; Consequential validity; Ecological validity; Generalizability; Measurement error; Precision; Accuracy; Stability; Sensitivity to change; Responsiveness to change; Standard error of measurement; Minimal detectable change; Minimal clinically important difference; Feasibility; Usability; Acceptability; Scalability; Generalizability theory; Classical test theory; Item response theory; Differential item functioning; Item banking; Computer-adaptive testing; Dimensionality; Heterogeneity; Homogeneity; Redundancy

Item S3: Inclusion selection questions

S. No	Questions
1.	Does the study investigate the psychometric properties of an instrument used to measure caregiver burden in patients with advanced kidney disease?<br>Yes ☐ Go to the next question ☐ Reject
2.	Is the study a primary research study, such as an original research article or a systematic review?<br>Yes ☐ Go to the next question ☐ Reject
3.	Is the sample size of the study large enough to provide a robust evaluation of the psychometric properties of the instrument?<br>Yes ☐ Go to the next question ☐ Reject
4.	Are the inclusion and exclusion criteria for the sample clearly stated?<br>Yes ☐ Go to the next question ☐ Reject
5.	Is the study design appropriate for evaluating the psychometric properties of the instrument?<br>Yes ☐ Go to the next question ☐ Reject
6.	Are the results of the study clearly reported, including statistics and sample demographics?<br>Yes ☐ Go to the next question ☐ Reject
7.	Is the study published in a peer-reviewed journal?<br>Yes ☐ Go to the next question ☐ Reject
8.	Is the study written in English?<br>Yes ☐ Go to the next question ☐ Reject
9.	Are the items or questions included in the instrument clearly described?<br>Yes ☐ Go to the next question ☐ Reject
10.	Are the results of the psychometric testing presented in such a way that the specific psychometric properties of the instrument can be evaluated?<br>Yes ☐ Go to the next question ☐ Reject
11.	Are the results of the study representative of the population of interest?<br>Yes ☐ Go to the next question ☐ Reject
12.	Does the study provide a detailed description of the methodology used to evaluate the psychometric properties of the instrument, including details of the statistical analysis?<br>Yes ☐ Go to the next question ☐ Reject

13.	Has the instrument been tested in a real-world setting or in a clinical trial?<br>Yes ☐ Go to the next question ☐ Reject
14.	Is the instrument reported to be used in a clinical setting or in research setting?<br>Yes ☐ Go to the next question ☐ Reject

Item S4: Data extraction form

Study Characteristics	Data to be Extracted
Study identification	Author(s), Year of publication, Title, Journal, Country, DOI, PMID, Funding sources, Conflicts of interest, Ethical clearance, Study registration
Study design	Study type (e.g., cross-sectional, prospective, observational, experimental), Sample size, Recruitment method (convenience, random, consecutive), Inclusion/exclusion criteria, Duration of follow-up, Data collection method (e.g., self-reported, interview-administered, multi-method), Data analysis method (e.g., descriptive, inferential statistics), Statistical analysis software used, Power calculation (if done)
Participants	Number of participants, Demographic characteristics (e.g., age, gender, ethnicity, stage of CKD, dialysis modality), Comorbidities, Caregiving experience (duration, frequency, type of care given), Relationship to the patient (e.g., spouse, child, parent, other), Socioeconomic status, Education level, Employment status, Quality of life, Patient's health status
Instrument	Name, Number of items, Response format (e.g., Likert scale, visual analogue scale, categorical), Administration method (e.g., self-reported, interview-administered), Language, Translation and cultural adaptation (if applicable), Reference, Cut-off scores (if applicable), Score range, scoring method, Score interpretation, Test-retest reliability, Internal consistency, Concurrent validity
Measurement properties	Reliability (e.g., test-retest, internal consistency, inter-rater), Validity (e.g., content, criterion-related, construct, known-groups), Responsiveness (e.g., minimal detectable change, effect size, standardized response mean), Feasibility (e.g., time required to complete, ease of administration), Practicality (e.g., cost, availability), Cultural sensitivity, Norms, floor and ceiling effect
Quality assessment	Risk of bias (COSMIN checklist), Quality of the measurement properties (e.g., scores for reliability, validity, and responsiveness)
